# Effects of microenvironment in osteosarcoma on chemoresistance and the promise of immunotherapy as an osteosarcoma therapeutic modality

**DOI:** 10.3389/fimmu.2022.871076

**Published:** 2022-10-13

**Authors:** Lei Yu, Jian Zhang, Yunfeng Li

**Affiliations:** Department of Radiation Oncology, The Second Affiliated Hospital of Jilin University, Changchun, China

**Keywords:** Osteosarcoma, tumor microenvironment, chemoresistance, immunotherapy, immune checkpoint inhibitors

## Abstract

Osteosarcoma (OS) is one of the most common primary malignant tumors originating in bones. Its high malignancy typically manifests in lung metastasis leading to high mortality. Although remarkable advances in surgical resection and neoadjuvant chemotherapy have lengthened life expectancy and greatly improved the survival rate among OS patients, no further breakthroughs have been achieved. It is challenging to treat patients with chemoresistant tumors and distant metastases. Recent studies have identified a compelling set of links between hypoxia and chemotherapy failure. Here, we review the evidence supporting the positive effects of hypoxia in the tumor microenvironment (TME). In addition, certain anticancer effects of immune checkpoint inhibitors have been demonstrated in OS preclinical models. Continued long-term observation in clinical trials is required. In the present review, we discuss the mutualistic effects of the TME in OS treatment and summarize the mechanisms of immunotherapy and their interaction with TME when used to treat OS. We also suggest that immunotherapy, a new comprehensive and potential antitumor approach that stimulates an immune response to eliminate tumor cells, may represent an innovative approach for the development of a novel treatment regimen for OS patients.

## 1 Introduction

Osteosarcoma (OS) is an osteoid-producing malignancy of mesenchymal origin. Worldwide incidence is 3.4 cases per million people per year (1). OS (accounting for 56%) is much more common than Ewing sarcoma, chondrosarcoma, and chordoma (2). Primary OS affects children, teenagers, and elders, with age-specific incidence varying according to histological subtype (**Table 1**). OS typically affects patients aged 10–30 years. In the group aged 25–59 years, the male to female incidence ratio of OS is 1.28:1 and the number is elevated (1.43:1) in the group aged 0–24 years. In addition, the ratio varies in diverse populations (3). OS frequently arises in the long bones (particularly the distal femur or the epiphysis of the proximal tibia) (3, 4). OS carcinogenesis is a complex process involving genetic mutations and dysregulation of epigenetic pathways (5). However, through whole-genome and exome sequencing, transcriptome assessment of gene expression, and epigenetic modifications, it was revealed that there was remarkable genomic complexity and significant inter-patient heterogeneity of genes in OS samples (6).

**Table 1:** Categories and treatment options for OS.

Subtype of OS	Incidence	Common anatomical distribution	Chemosensitivity	Radiosensitivity
Conventional OS (3 subtypes: osteoblastic, chondroblastic, fibroblastic)	75.0%	Metaphysis of long bone around knee and shoulder joint, axial skeleton	Sensitive	Radiotherapy can be useful
Parosteal OS	3.5–4%	Posterior cortex of distal femur	Hyposensitivity	Hyposensitivity
Telangiectatic OS	3–4%	Similar to conventional OS	Sensitive	Hyposensitivity
Periosteal OS	1.5–2%	Tibia or femur	Hyposensitivity	Hyposensitivity
Small cell OS	1.5%	—	Sensitive	Sensitive
Low grade central OS	1–2%	Intramedullary distal femur, proximal tibia, pelvis	—	—
High-grade surface OS	<1%	Long bone, distal femur	Sensitive	Radiotherapy can be useful

Currently, the standard treatment protocol for patients with OS comprises extensive surgical resection, radiotherapy, and administration of chemotherapeutic agents. The current curative regimen combines surgery with multiple modes of chemotherapy using several cytotoxic agents, such as cis-platinum, doxorubicin, high-dose methotrexate, and ifosfamide during preoperative and postoperative periods (7). Surgical excision is preferred over systemic therapy for recurrent disease while unresectable cases would be treated by systemic therapy or comprehensive therapy (8). *Via* radiotherapy, we can take advantage of ionizing radiation to help eliminate microscopic or minimal residual disease in situations where substantial surgical resection is not feasible (9). However, in the majority of OS cases, the efficacy of radiotherapy is limited, and the indications for this approach are finite (10). Despite aggressive interventions, patient outcomes have not significantly improved over the last 20 years. During this period, the well-known phenomenon of chemotherapeutic resistance has prevented improvements in prognosis (7).Furthermore, OS prognosis has not improved over the past several decades. Facing these hindrances to current curative regimens, identifying novel therapeutics is critical to promote the management of OS.

Multidrug resistance is a difficult problem that results in unsatisfactory clinical outcomes (11). In recent years, many studies have demonstrated that the tumor microenvironment (TME) appears to influence clinical outcome and therapeutic response by regulating tumor chemoresistance (12, 13). Managing TME-related drug resistance may profoundly affect cancer therapeutic strategies. TME-related multidrug resistance can be mediated by hypoxic conditions and soluble factors secreted by tumors or stromal cells. Inhibiting extracellular ligand–receptor interactions and downstream pathways are among the TME-targeted treatment methods (13). We propose that focusing on the primary mechanism of TME-related multidrug resistance would yield substantially greater benefits. A combination of drugs that can simultaneously attack tumor cells and the TME may help reduce chemoresistance. Herein, we review the effects and mechanisms of chemoresistance regulated by the OS TME through hypoxia and immune cells. This review also suggests the novel and therapeutic potential of immunotherapy for the management of OS treatment.

There is a pressing need to investigate novel therapies that could impact OS because of its resistance to chemotherapy. Immunotherapy has gained considerable attention since it has demonstrated efficacy in the treatment of cancers. For instance, the combination of nab-paclitaxel and atezolizumab was recently approved by the Food and Drug Administration (FDA) for patients with unresectable locally advanced or metastatic TNBC whose tumors express PD-L1 based on a PFS benefit over chemotherapy in the Impassion130 trial (14). Interactions between TME modulation and the immune system may enhance therapeutic efficacy. Immunotherapy is a promising therapeutic strategy for improving the curative efficacy of existing OS treatments despite chemoresistance. In the current review, we present the mechanism of TME-related chemoresistance and describe the modulatory effects of the TME in OS treatment. Subsequently, we discuss new technologies and strategies— immunotherapy that can be adapted to explore the roles of the TME in improving the curative effects of drug treatment by modifying TME-associated factors. A better understanding of the molecular mechanisms of immunological therapy is required, as current research suggests that this may be a more promising method to develop and implement optimal preventive and curative approaches to treating patients with OS. Our review of the active mechanisms of immune-cell regulation within the TME and the impressive clinical results achieved by stimulating antitumor immune responses supports the implementation of immunotherapy together with anticancer therapies for the treatment of OS.

## 2 Mechanisms of TME-mediated chemotherapy resistance in OS

The TME is composed of multiple cell types (fibroblasts, endothelial cells, and immune cells), extracellular components that surround tumor cells and are nourished by the vasculature (chemokines, cytokines, hormones, and ECM), and various physical and chemical factors surrounding tumor cells (hypoxia and acidic environment) (15). The TME plays a pivotal role in carcinogenesis, tumor development, and metastasis. For example, the TME makes a remarkable contribution to the acquisition and maintenance of cancer hallmarks, such as inducing angiogenesis, sustaining proliferative signaling, resisting cell death, and activating invasion and metastasis (15). The TME also exerts profound effects on therapeutic efficacy. TME-reduced multidrug resistance results from sustained crosstalk between tumor cells and their surrounding matrix. Owing to genomic instability, tumor cells are prone to chemoresistance, whereas non-tumor cells in the TME are more genetically stable and susceptible to stimulation. Hence, the insight that cancer progression and therapeutic resistance are closely related to the TME raises the possibility that efforts devoted to targeting TME elements or their signaling pathways could achieve therapeutic advances for cancer patients.

### 2.1 Hypoxic TME and chemoresistance in OS

Tumor cells typically live in a state of hypoxia because of hypermetabolism, abnormal proliferation, and high oxygen consumption (16). A compelling set of links between drug resistance and hypoxia-inducible factors (HIFs) has emerged (17). Following hypoxia, HIFs secreted for hypoxic adaptation are capable of triggering the expression of a variety of genes related to erythropoiesis, glycolysis, and angiogenesis, as well as restore oxygen homeostasis at the epigenetic and transcriptional levels (18, 19). Undoubtedly, hypoxia may result in an acidic environment and the Warburg effect is the typical example: tumor cells tend to obtain energy through glycolysis. Through H+-ATPases, Na+-H+ exchangers, and HCO3- transporters, the acidoid can be transported from an intracellular area to an extracellular one (20, 21). In addition, the rapid tumor proliferation and abnormal vascular structures accelerate further accumulation of acid, eventually leading to an extracellular pH of 6.7–7.1 for tumor cells and an intracellular pH > 7.4. In comparison, the extracellular and intracellular pH of normal cells is approximately 7.4 and 7.2, respectively (12).

#### 2.1.1 Hypoxic TME induces chemoresistance by regulating signaling pathways

Accumulating evidence suggests that hypoxia plays a vital role in the molecular mechanisms underlying drug-resistant cancers by regulating gene expression (**Table 2**). For instance, overexpression of efflux transporters (primarily the ATP-binding cassette [ABC] superfamily of pump proteins, including P-glycoprotein [P-gp] encoded by the multidrug resistance gene 1 [MDR-1]) may amplify the efflux of certain drugs from tumor cells, thereby resulting in resistance to anticancer drug (37–39). Roncuzzi et al. (35) showed that hypoxia-inducible factor 1-alpha (HIF-1α), the most influential regulator of cell adaptation to hypoxia, promotes export of intracellular doxorubicin by increasing the level of P-gp in OS. Furthermore, by modulating the expression of c-Myc and p21, HIF-1α can prevent doxorubicin-induced OS apoptosis, indicating that HIF-1α could be a valuable therapeutic target. Ma et al. (40) determined that overexpression of spindle-and kinetochore-associated complex subunit 1 (SKA1) can reduce expression of some multidrug resistance genes, such as *ABCB1 (MDR1)*, *ABCC2 (MRP2)*, and *GSTP1*, as well as enhance sensitivity to the drugs epirubicin and ifosfamide, which have been used in OS patients. Downregulation of SKA1 expression is mediated by hypoxia, which increases chemoresistance in human OS cells. Li et al. (32) concluded that hypoxia and the Notch signaling pathway display crosstalk. Specifically, hypoxia upregulates the Notch signaling pathway in human OS cells, contributing to OS cell proliferation and G0/G1-S-G2/M phase transition and consequently promoting multidrug resistance. Western blot analysis showed hypoxia elevated secretion of HIF-1α and Notch1, resulting in the upregulation of *MRP1* (which encodes a homolog of the multidrug resistance protein).

**Table 2:** A schematic diagram of the expression of hypoxic and drug resistance factors.

Study. (year). Ref	Source	Mechanism	Target gene	Expression change	Clinic characters relatedness
Wang et al.(2019) (22)	MG-63 and U2-os cells	Visfatin was involved in cisplatin resistance of OS cells by upregulating expression of Snail via HIF-1α induced transcription	Snail and its mRNA	↑	cisplatin resistance
Keremu et al.(2019) (23)	20 osteosarcoma patient samples and human OS cell lines (MG-63, U-2OS and SaoS-2)	Overexpression of miR-199a resensitizes cisplatin resistant cells to cisplatin through inhibition of HIF-1α	miR-199a	↑	cisplatin resistance
Zheng et al.(2017) (24)	U-2OS (derived from bone tissues of a 15-year-old OS patient) and MG-63 (derived from bone tissues of a 14-year-old OS patient) cells	HIF-1α-induced Mxd1 up-regulation suppresses the expression of PTEN under hypoxia, which leads to the activation of PI3K/AKT antiapoptotic and survival pathway	Mxd1	↑	hypoxia-induced cisplatin resistance
Guo et al.(2017) (25)	MG63, U2OS and 143B cells	MiR-335 targets CSCs and regulates OS stem cell-like properties via downregulated POU5F1 to synergize with chemotherapeutic drugs	miR-335	↓	stem cell-like properties
Ma et al.(2017) (26)	Human OS cells (SOSP-9607, MG-63, SaOS-2)	Hypoxia increased the expression of MRG and enhanced the sensitivity of EPI and IFO in OS patients	SKA1	↓	chemotherapy resistance
Zhao et al.(2016) (27)	MG-63 and U2-os cells	Hypoxia reduced sensitivity to Dox by promoting the AMPK signaling and has no association with HIF-1α	AMPK	↑	Dox resistance and Dox-induced apoptosis
Zhou et al.(2016) (28)	human OS cell lines (MG-63, U-2OS and SaoS-2)	Hypoxia induced microRNA-488 expression to promote proliferation, reduce apoptosis and decrease the Dox sensitivity of OS cells	microRNA-488	↑	tumor proliferation, apoptosis and Dox resistance
Wang et al.(2016) (29)	human OS cell lines MG-63, U2OS, Saos-2 and normal os- teoblastic cell line HOB	miR-367 suppressed the increase of KLF4 induced by ADR in OS cells, as well as Bax and cleaved caspase-3	MiR-367	↑	ADR-induced apoptosis
Lin et al.(2016) (30)	U2OS and G293 cell lines	miR-202 promotes chemotherapy resistance by targeting PDCD4	miR-202	↑	Dox resistance and Dox-induced apoptosis
Xu et al.(2016) (31)	MG-63 cell line and Dox-resistant cell line (Mg-63/Dox)	miR-30a downregulated in Mg-63/Dox and miR-30a reduced chemoresistance via suppressing Beclin-1-mediated autophagy	miR-30a	↓	chemoresistance and autophagy
Li et al.(2016) (32)	human MG-63 OS cells	Notch signaling is up-regulated in human OS cells under hypoxia and Notch1 may represent a viable target to overcome chemoresistant OS cells in a hypoxic niche by regulating MRP1 gene expression.	Notch1 and MRP1	↑	chemoresistant
Guo et al.(2015) (33)	human MG-63 OS cells	HIF-1α inhibitor combined with paxilitaxel blocked autophagy and augmented the anti-tumor effects.	—	—	paxilitaxel-induced apoptosis
Zhang et al.(2015) (34)	human OS cell lines (MG-63 and U-2OS)	miR-301a and HMGCR were up-regulated in chemotherapy-resistant OS, subsequently reduced Dox-induced cell apoptosis and contributed to chemoresistance of OS cells	miR-301a	↑	Dox resistance and Dox-induced apoptosis
Roncuzzi et al.(2014) (35)	human MG-63 OS cells	HIF-1α hindered Dox-induced apoptosis and promoted the outward transport of intracellular Dox by activating P-gp expression in OS in normoxic conditions	c-Myc	↓	Dox-induced apoptosis
			p21	↑	Dox-induced apoptosis
			MDR-1/P-gp	↑	Dox resistance
Scholten et al.(2014) (36)	Human OS cells (143B, MNNG/HOS, MG-63)	Hypoxic OS cells can be sensitized to Dox treatment by inhibition of the Wnt/β-catenin signaling pathway	Wnt/β-catenin signaling pathway	↓	Dox-mediated toxicity

*CSCs, cancer stem cells; ADR, adriamycin; Dox, Dox;↑, upregulated; ↓, downregulated.

Another mechanism of hypoxic TME function was reported by Zhao et al. (27). Hypoxia visibly impaired the sensitivity of U2-OS cells to doxorubicin by upregulating the AMPK signaling pathway. This impaired sensitivity was independent of HIF-1α but was promoted by hypoxia in U2-OS cells. Further research (27) has confirmed that the primary mechanism is associated with a distinct upregulation of phosphorylated AMPK and phosphorylated acetyl-CoA carboxylase (ACC). Both were modulated by the AMPK activator AICAR and the AMPK inhibitor Compound C. AICAR and Compound C decreased or increased the sensitivity of U2-OS cells to doxorubicin by promoting or downregulating AMPK activity, respectively. Therefore, the prevalent application of HIF inhibitors in clinical settings remains controversial, despite progress made in the research of many types of tumors (41).

#### 2.1.2 Hypoxic TME induces chemoresistance by regulating autophagy

Autophagy, also known as type II programmed cell death, is a self-digestion process by which cells form double-membraned autophagic vesicles that sequester damaged, denatured, or senescent organelles, and target them for degradation in lysosomes (42). The complicated relationship between autophagy and carcinoma indicates that it plays a dual role in tumorigenesis and tumor development (43). In the early stages of tumorigenesis, the inhibition of autophagy promotes cell proliferation, indicating that this process plays an inhibiting role in the earliest stages of tumor development. Later in tumor development, autophagy inhibits tumor cell apoptosis and promotes metastasis, allowing tumor cells to continue proliferating. Increasing evidence supports that autophagy can cope with intracellular and environmental stresses, such as hypoxia or nutrient shortage, thereby favoring tumor progression (42, 44). For instance, the ATG4B chemical inhibitor (a cysteine proteinase that activates LC3 which is crucial for OS development) may result in autophagy deficiency and a decreased proliferation *in vitro* and tumor growth *in vivo* (45). This indicates that autophagy is capable in promoting proliferation and resistance to anti-cancer therapy in OS tumor cells (46, 47). As a result, tumor cells can survive under conditions of hypoxia or nutrient deficiency *via* autophagy in advanced stages of tumor development. A recent study by Moscowitz et al. (48) suggests that hypoxia could promote resistance to irradiation by activating autophagy to accelerate the clearing of reactive oxygen species (ROS) in MG-63 human OS cells. These hypoxia-exposed OS cells displayed compartmental recruitment of GFP-tagged LC3 and restored the radiation sensitivity on autophagy inhibition, showing the possible causative link between hypoxia and autophagy. The regulating function does not just apply to radiotherapy-resistance. Zhang et al. (49) showed that CD271+ OS cells showed a higher autophagy activity than CD271- OS cells under hypoxia while autophagy deficiency in the CD271+ cells restored chemotherapeutic sensitivity and restricted the advantage of CD271+ OS cells in terms of tumorigenesis *in vivo*. Additionally, autophagy can promote tumor cell growth by inducing angiogenesis (50).

In contrast, autophagy can protect tumor cells from the damage of chemotherapy and/or radiotherapy; however, it can induce programmed apoptosis of tumor cells in response to antineoplastic drugs. Therefore, the complicated role of autophagy in tumor treatment is bidirectional and has been examined by a growing number of scholars. The results of a recent study (33) suggested that paxilitaxel and a HIF-1α inhibitor can be used to effectively improve OS chemotherapy in the future. This study illustrates that PTX induces autophagy through the HIF-1α pathway. Moreover, in rescue studies, co-treatment with the HIF-1α inhibitor YC-1 and autophagy inhibitor 3-methyladenine markedly blocked autophagy and blunted PTX resistance (33).

#### 2.1.3 Hypoxic TME induces chemoresistance by modulating microRNAs

There is evidence that microRNA (miRNA) dysregulation is predictive of tumor progression and prognosis and contributes to tumorigenic processes (**Table 2**). HIF-1α has been identified as a direct target of miRNAs in multiple tumor types. For instance, the overexpression of miR-199a re-sensitizes cisplatin-resistant cells by inhibiting the HIF-1α pathway *in vitro* and *in vivo* (23). Furthermore, exogenous overexpression of miR-488 induced proliferation and suppressed sensitivity to doxorubicin in OS cells by targeting the tumor suppressor BIM (BH3-only protein, a mediator of apoptosis). Hypoxia can induce expression of miR-488, which is present in high concentrations in primary OS tissues and OS-derived cells, by binding to the hypoxia response element (HRE) in its promoter (28).

### 2.2 Immune cells within the TME modulate chemoresistance in OS

At the onset of carcinogenesis, immune cells infiltrate the TME. Intriguingly, the dynamic tumor immune landscape has a profound impact on tumor development and dissemination, and the activation state of immune cells within the TME can fluctuate.

#### 2.2.1 Tumor-associated macrophages modulate chemoresistance in OS

Tumor-associated macrophages (TAMs) are key components of the TME and in most cases display tumor-suppressive properties and therapeutic response regulations. In solid tumors, TAMs are rooted in circulating monocytes rather than in proliferating resident macrophages within tumors. Monocytes in the bone marrow can enter neoplasms *via* the bloodstream and subsequently differentiate into macrophages. Based on their polarization condition, macrophages are classified as type M1 or M2. M1 macrophages differentiate in response to the Th1 cytokine interferon-gamma (IFNγ), whereas M2 macrophages are activated by Th2 cytokines, such as interleukin (IL)-4, IL-10, and IL-13 (51, 52). Similarly, M1 macrophages are generally considered to be cancer-fighting, while M2 macrophages promote carcinogenesis (53, 54). In fact, the TME plays a major regulatory role in the functional polarization of TAMs (54).

Chemotherapeutic agents may induce misdirected repair responses orchestrated by TAMs, contributing to limiting tumoricidal efficacy in drug applications (55). Compelling evidence has revealed that TAMs can mediate resistance to certain chemotherapeutics (5-fluorouracil, doxorubicin, paclitaxel, and platinum salts) and anti-VEGF (vascular endothelial growth factor) treatment *in vitro* and *in vivo* (56–59). Multiple mechanisms underlie the contribution of TAMs to chemoresistance: (i) several chemokines secreted by tumor cells increase the recruitment of immunosuppressive TAMs and suppress CD8^+^ T cell responses during chemotherapy (60); (ii) TAMs develop the capacity to create a number of inhibitory cytokines, such as IL-1β, IL-6, IL-10, and TGF-β, consequently blocking the activation of an effective adaptive response and leading to T cell suppression in the TME (51, 61); (iii) TAM-derived cathepsins may mediate the activation of the nuclear factor-kappa B (NF-κB) signaling pathway and the signal transducer and activator of transcription 3 (STAT3) to facilitate therapeutic resistance (62–64); (iv) TAMs increase the tumor initiating potency of cancer stem cells (CSCs) and preserve CSCs from chemotherapy damages, thereby blunting chemotherapeutic responses (64); (v) by upregulating the enzyme cytidine deaminase that metabolizes the drug following its transport into cancer cells, TAMs can produce acquired resistance to chemotherapy (65).

Specifically, TAMs can activate STAT3, promote epithelial-mesenchymal transition (EMT), and upregulate matrix metallopeptidase 9 (MMP-9) in OS cells to facilitate chemoresistance. Evidence verified in animal models and OS patients demonstrated that TAMs possess the ability to induce OS cell migration and invasion by upregulating cyclooxygenase-2 (COX-2) and MMP9, phosphorylating STAT3, and promoting EMT (66). Shao et al. discovered that M2 TAMs enhanced the tumor initiation and stem-like capacity of CSCs by upregulating the number of CD117(+)Stro-1(+) cells accompanied by an increase in CSC markers (CD133, CXCR4, and Oct4) (67). This indicates that M2 TAMs induce OS cells to acquire stem cell characteristics and subsequently enhance the drug resistance of OS. Furthermore, evidence from this study suggest that the ratio of M1 to M2 macrophages could transform the OS chemoresistance by regulating the TME. Taken together, there is a growing interest in TAM-centered treatment regimens, which involve converting TAM-polarization from an M2 to M1 phenotype in the TME, transporting anticancer drugs into the TME *via* TAMs, suppressing the recruitment of monocytes and TAMs, and neutralizing the original tumor products of TAMs (68).

Based on the crucial role that TAMs play in OS growth and metastasis, many clinical trials were moved forward (**Table 3**). For instance, the use of mifamurtide (the liposome-encapsulated muramyl and macrophage-activating agent) as an effective immunomodulatory can greatly improve the event-free survival rate, suppress tumor proliferation, and induce cell differentiation by switching TAM-polarization from an M2 phenotype to M1 in patients with OS (69–72). Induced by IFN-γ, mifamurtide can activate macrophages to exert antitumor activities (73). In a phase II clinical trial, mifamurtide combined with chemotherapeutics (cisplatin, doxorubicin, methotrexate, and ifosfamide) promoted the elevation of the overall survival rate and progression-free survival (PFS) rate through the infiltration of activated macrophages in the adolescent OS group (71). To remodel the immune response, mifamurtide has been ratified by the European Medical Agency for the adjuvant chemotherapy of nonmetastatic OS (74). Additionally, the specific blocking of receptor-ligand binding between macrophages and OS cells may improve phagocytosis and antitumor effects of macrophages, and appears to be a promising strategy for cancer therapy. Colony-stimulating factor 1 receptor (CSF1R), which is capable of controlling the differentiation and survival of macrophages and is related to the prognosis of OS, can be selectively suppressed by pexidartinib (a novel small molecule tyrosine kinase inhibitor) (75, 76). Pexidartinib depletes TAMs and boost antitumor immune responses by blocking CSF1R and has been identified to be safe and well-tolerated in anti-cancer therapy (77, 78). It is currently being recruited for unresectable OS patients who are treated with pexidartinib combined with sirolimus (NCT02584647). In addition, the α4-integrin located on the surface of TAMs is able to bind to vascular cell adhesion molecule 1 (VCAM-1) that is expressed in the OS cytomembrane, resulting in the significant protection of OS cells from pro-apoptotic cytokines (79). Therefore, it would be effective to prevent tumor proliferation and metastasis in OS by using antibodies that are directed against α4-integrin, such as natalizumab (NCT03811886) (80).

**Table 3:** Schematic diagram of progressive clinical trials on OS TAM-centered treatments.

Clinical trial	Phase	Combined drug	Interventions	Therapeutic target
NCT02441309	II	Ifosfamide + Mifamurtide	Group 1: mifamurtide alone; Group 2: ifosfamide alone for 6 weeks then ifosfamide + Mifamurtide for 6 weeks, then mifamurtide alone for 30 weeks; Group 3: ifosfamide + mifamurtide for 12 weeks then mifamurtide alone for 24 weeks. All participants will receive 36 weeks or more of mifamurtide.	Macrophage
NCT00631631	—	—	Mifamurtide (L-MTP-PE), intravenous, at a dose of 2 mg/m^2 twice weekly (at least 3 days apart) for 12 weeks, and then weekly for an additional 24 weeks, for a total of 48 doses in 36 weeks.	Macrophage
NCT03811886	I	Natalizumab	Traditional 3 + 3 escalation of natalizumab at a weight-based dosing 2 mg/kg not exceeding 300 mg. If no subjects experience a dose limiting toxicity (DLT), 3 more subjects are enrolled at the next dose of 3 mg/kg, not to exceed 300 mg. If no subjects experience a DLT, 3 more subjects will be enrolled at the next and final dose of 4 mg/kg, not exceeding 300mg.	TAMs
NCT01459484	II	Methotrexate, Cisplatinum, Doxorubicine, Ifosfamide + Mifamurtide	Group1: Chemotherapy for patients who over express ABCB1/P-glycoprotein:PRE-SUGERY TREATMENT: methotrexate:12 g/m2 (3cycles) + cisplatinum:120 mg/m2 (3 cycles), doxorubicin + ADM 75 mg/m2 (3 cycles); POST-SURGERY TREATMENT for good responder patients with positive PGLYCOPROTEIN:methotrexate 12 g/m2 (10 Cycles) cisplatinum 120 mg/m2; Doxorubicin 90 mg/m2 MEPACT 2 mg/m2 twice a week for the first 3 months the weekly for the next 6 months (total length of treatment: 44 weeks); POST-SURGERY TREATMENT for poor responder patients with positive P-GLYCOPROTEIN: methotrexate 12 g/m2; cisplatinum 120 mg/m2; doxorubicin 90 mg/m2, ifosfamide 15 g/m2 MEPACT 2 mg/m2 twice a week for the first 3 months the weekly for the next 6 months (total length of treatment 44 weeks);Group 2: high-grade osteosarcoma treatment for patients who do not over express ABCB1/P-glycoprotein: high-grade osteosarcoma that does not over express ABCB1/P-glycoprotein will be treated with a standard 3-drug regimen PRE-SUGERY TREATMENT: methotrexate: 12 g/m2 (3 cycles), cisplatinum: 120 mg/m2 (3 cycles) doxorubicin: ADM 75 mg/m2 (3 cycles) POST-SURGERY TREATMENT: methotrexate 12 g/m2 (10 cycles), cisplatinum 120 mg/m2; doxorubicin 90 mg/m2 (total length 34 weeks)	TAMs
NCT02584647	I	Sirolimus + PLX3397	Subjects with unresectable or metastatic sarcoma will take orally PLX3397 (600 - 1000mg) in combination with Sirolimus (2-6 mg) daily	TAMs
NCT02502786	II	GM-CSF + humanized anti-GD2 antibody: hu3F8	One cycle consists of treatment with hu3F8 at a dose of 2.4 mg/kg/dose for 3 days (day 1, 3, and 5) in the presence of subcutaneous (sc) GM-CSF (day 4 through 5). These 3 doses of hu3F8 and 10 days of GM-CSF constitute a treatment cycle. Cycles are repeated at ~2–4-week intervals between first days of hu3F8, through 5 cycles.	GM-CSF

Owing to the antitumor effects of macrophages in tumorigenesis, the application of immunomodulatory therapy is gaining increased attention. A variety of macrophage-related immune checkpoint inhibitors (ICIs) have been found to inhibit the proliferation and metastasis of OS through TAMs (**Table 3**). For instance, the transmembrane protein CD47, which is overexpressed in human OS samples, is an innate immune checkpoint and binds to the inhibitory receptor signal regulatory protein α (SIRPα) on the surface of TAMs, playing roles in the evasion of phagocytosis and cell mortality (81–84). Preclinical studies have indicated that CD47 may be a potential therapeutic target in OS treatment. The anti-CD47 monoclonal antibody may enhance the phagocytic effects of macrophages by restraining the interaction between CD47 and SIRPα in OS mouse models (84, 85). The efficacy of CD47 mAb + doxorubicin therapy demonstrates visibly increased TAM levels and their further phagocytic capabilities in mouse models of OS, resulting in an additive therapeutic effect (86). It was also confirmed that SIRPα knockout macrophages boost phagocytosis in an OS-bearing mice model (87). Although clinical trials are performed with CD47/SIRPα blocking on multiple malignancies, such as B-cell lymphomas (NCT02953509), acute myeloid leukemia (NCT05266274), non-small cell lung cancer (NCT04881045), there are currently no ongoing registered clinical trials in OS using this concept. However, even without CD47 targeting drugs in OS therapy, these suggested strategies targeting CD47/SIRPα may still be an efficient treatment strategy in patients with OS (88).

#### 2.2.2 Myeloid-derived suppressor cells modulate chemoresistance in OS

Myeloid-derived suppressor cells (MDSCs) are consisting of myeloid progenitor cells, immature macrophages, immature granulocytes, and immature dendritic cells. These cells expand during carcinogenesis and significantly suppress T cell responses (89). The regulatory mechanisms of MDSCs are related to multiple immunosuppressive factors in suppressing T cell-mediated antitumor immunity, including the production of ROS, inducible nitric oxide synthase (iNOS), COX-2, TGF-β, and arginase (90–92). In return, tumor cells secrete COX-2 and prostaglandin E2 (PGE2) to provoke MDSCs expressing arginase and iNOS (93). Due to the novel focus of MDSCs as the target in OS immunotherapy, several studies have been highlighted (**Table 4**).

**Table 4:** A schematic diagram of promising therapeutic roles of MDSCs in OS.

Study. (year). Ref	Source	Mechanism	Promising therapeutic target
Ligon et al.(2021) (94)	tissue from OS patients	Targeting MDSCs suppressing T-cell infiltration into the PM of OS to block OS metastasis	Gene regulation
Deng et al.(2020) (95)	80 OS patients from database and 27 OS patients	Neoadjuvant chemotherapy reduce the MDSCs number and convert OS into an immune “hot” tumor.	MDSCs’ reduction
Jiang et al.(2019) (96)	K7M2 mouse OS model	OS-infiltrating MDSCs were CXCR4 positive and would migrate toward an SDF-1 gradient. The axis of CXCR4/SDF-1 could reduce the apoptosis of MDSCs.	MDSCs’ apoptosis induction
Shi et al.(2019) (97)	K7M2 mouse OS model	Combining SNA with anti-PD1 regulated innate immune cells, slowed OS tumor growth and prolonged survival time of tumor-bearing mice *via* inhibiting the function of MDSCs with a selective PI3Kδ/γ inhibitor to enhance responses to immune checkpoint blockade.	Supplement classical immunotherapy
Uehara et al.(2019) (98)	K7M2neo OS model	Met regulated the metabolism of MDSCs to decrease OXPHOS and enhance glycolysis to inhibit OS growth.	MDSCs’ metabolism
Guan et al.(2017) (99)	Mouse tumor model	IL-18 inducing MDSC to infiltrate into the OS parenchyma	MDSCs’ migration
Long et al.(2016) (100)	NSG mice	ATRA treatment enhances efficacy of GD2-CAR T cells against OS by eradicating monocytic MDSCs and diminishing the suppressive capacity of granulocytic MDSCs.	MDSCs’ reduction

For instance, Uehara et al. (98) found that metformin (Met) reduced the number of MDSCs in tumors, particularly polymorphonuclear MDSCs (PMN-MDSCs), which is independent of T cells. The molecular mechanism underlying this phenomenon involves decreased oxidative phosphorylation (OXPHOS) and increased glycolysis in the metabolism of MDSCs regulated by Met, suggesting that we should regard the regulation of metabolism of MDSCs as a potential therapeutic strategy. Additionally, the reduced reactive oxygen species (ROS) concentration and proton leakage in MDSCs and TAMs could be confirmed in the OS tumor model (98). Furthermore, to suppress T cell function, MDSCs not only remove the key nutrients for T cell proliferation and metabolism by freeing ROS, but also inhibiting the trafficking of CTLs into the tumor (101). A recent study (96) showed that OS tissues were infiltrated by MDSCs with the ability to inhibit CTL expansion. Moreover, MDSCs were CXCR4+, and migrated toward the stromal cell-derived factor-1 (SDF-1) gradient in the OS TME. The axis of CXCR4/SDF-1 may mediate reduced apoptosis of MDSCs by activating the downstream AKT pathway. The authors also note that the anti-PD-1 anti-body immunotherapy effect was strengthened by targeting CXCR4 in an OS murine model. Moreover, IL-18 induced MDSCs to infiltrate into the tumor parenchyma in an OS model, suggesting an IL-18 inhibitor as a potential strategy in MDSC-targeted immunotherapy in patients with OS (99). MDSCs play a crucial role in refractoriness to several chemotherapeutic agents, such as doxorubicin, cisplatin, and ifosfamide, which are standard treatments for OS (95, 102, 103).

A localized disease approach cure rate of nearly 70% is achieved, while a metastatic disease approach cure rate of less than 25% can be achieved. Hence, therapies that prevent OS metastasis are crucial to patients with OS. Using MDSC-targeted therapy for blocking OS metastasis may also be a possible treatment as MDSCs inhibit the infiltration of T-cells into the PMN, especially pulmonary metastasis.

Overall, both hypoxia and immune cells within the TME serve as basic modulators of OS chemoresistance. However, there is more involving the correlation between hypoxia and the immune landscape. Many scholars would like to further explore the impact and interplay of hypoxia and immunity within the TME.

### 2.3 Angiogenesis-mediated drug resistance in OS

The process of angiogenesis is complex, highly adaptive, and a hallmark of cancer, which is crucial for tumor growth, metastasis, and drug resistance. A variety of processes accompany angiogenesis, including endothelial cell proliferation, differentiation, migration, recruitment of smooth muscle cells, and maturation of blood vessels (104). An imbalance between pro- and anti-angiogenic signals in tumors can form an abnormal vascular network that typically displays dilated, convoluted, and hyperpermeable vessels, resulting in spatiotemporal heterogeneity in either tumor blood flow and oxygenation or increased tumor interstitial fluid pressure (105). Moreover, dysregulation of angiogenic and angiocrine activities can trigger altered bone homeostasis (106). The physiological consequences of these vascular abnormalities and the resultant microenvironment fuel tumor progression are conspicuous in the impaired efficacy of chemotherapy, radiotherapy, and immunotherapy (105). Apart from the influence of angiogenesis in hypoxia, acidity, and increased interstitial fluid pressure toward drug resistance, the abnormal vascular structure of OS also limits delivery of anticancer drugs (107). As chemotherapeutics must cross blood vessel walls and penetrate tumor tissues to kill cancer cells, anticancer drug distribution is asymmetrical. Therefore, a proportion of target tumor cells located proximal to tumor blood vessels receive a potentially lethal concentration of the cytotoxic agent (108). Consequently, the killing effect of the drug is limited.

Preclinical studies (109–112) of OS have shown that anti-angiogenic inhibitors transform the abnormal tumor vasculature into normal vasculature, characterized by attenuation of hyperpermeability, a normal basement membrane, increased vascular pericyte coverage, and a resultant decline in tumor hypoxia and interstitial fluid pressure. In return, the ameliorative vascular phenotype could favor the metabolic profile of the TME, delivery of chemotherapy agents, efficacy of radiotherapy and immunotherapy, and a diminution in metastatic cells shed by tumors into circulation in mice. Clinical trials (113–116) of targeted anti-angiogenic drugs have demonstrated that OS patients with a low OS vascularization phenotype have higher overall and relapse-free survival rates. Furthermore, patients with a low OS vascularization phenotype showed a better response to neoadjuvant chemotherapy than that of other patient groups.

Although combinatorial regimens of anti-angiogenic drugs and chemotherapeutic agents have been widely accepted, several clinical studies (117, 118) found that these combinations yielded unsatisfactory results. For instance, the observed histological response and event-free survival rate in a phase II trial did not support further evaluation of the combination of chemotherapy and bevacizumab in OS (119). This may be due to the anti-angiogenic therapy itself. Although the abnormal structure and function of cancerous vasculature leads to an anoxic microenvironment and increases the difficulty of drug delivery, it is one of the main routes for immune cells as well as chemotherapy agents to travel through the blood vessels. Hence, the inhibition of vascular production affects the delivery and final efficacy of anticancer drugs.

Notably, cells and structures integrated within the TME strongly shape the functions of one another, modulating antitumor therapy. For instance, pre-existing blood vessels fail to perfuse the tumor sufficiently during tumor growth; thus, a microenvironment deficient in oxygen and nutrients is formed where metabolites and immunosuppressive modulators accumulate (120). The resultant anoxic microenvironment stabilizes HIF-1α or HIF-2α, subsequently activating PDK1 and LDH-A, promoting an acidic extracellular environment (121, 122). Furthermore, HIF-regulated vascular endothelial growth factors can induce angiogenesis (121). In addition, hypoxia alters cellular metabolism and regulates expression of several immunomodulatory molecules, thereby influencing the infiltration and phenotype of immune cells (122–124). Other hypoxia-driven signals affect immune cells as well, such as acidic environments, cytokines, and nutrient fluctuations. Thus, it seems that there is a complex and powerful relationship among anoxic and acidic environments, the tumor vascular system, and immune cells, orchestrating cellular progression and metastasis, ultimately leading to drug resistance (125).

All TME components mentioned above play important roles in drug resistance in OS therapy. Given the barriers involved in chemoresistance, novel therapeutic approaches to treat OS is urgently needed. In the present review, we summarize the effects and mechanisms of the TME in terms of chemoresistance in OS. Moreover, we pay considerable attention to immune cells, a key component of the TME, as a valid strategy to address drug resistance due to the clinical success of emerging ICIs in immunotherapy. A detailed analysis of other popular treatment regimens is beyond the scope of this manuscript. Thus, suggest that those interested in reading other comprehensive reviews to find them elsewhere (126, 127). The current review highlights the therapeutic potential of immunotherapy in the management of OS. Herein, we review recent advances in promising new immune checkpoint targets for their use in the improvement of chemoresistance and treatment effects in OS therapy.

## 3 Immunotherapy: A promising therapeutic option for OS

**Figure 1:**
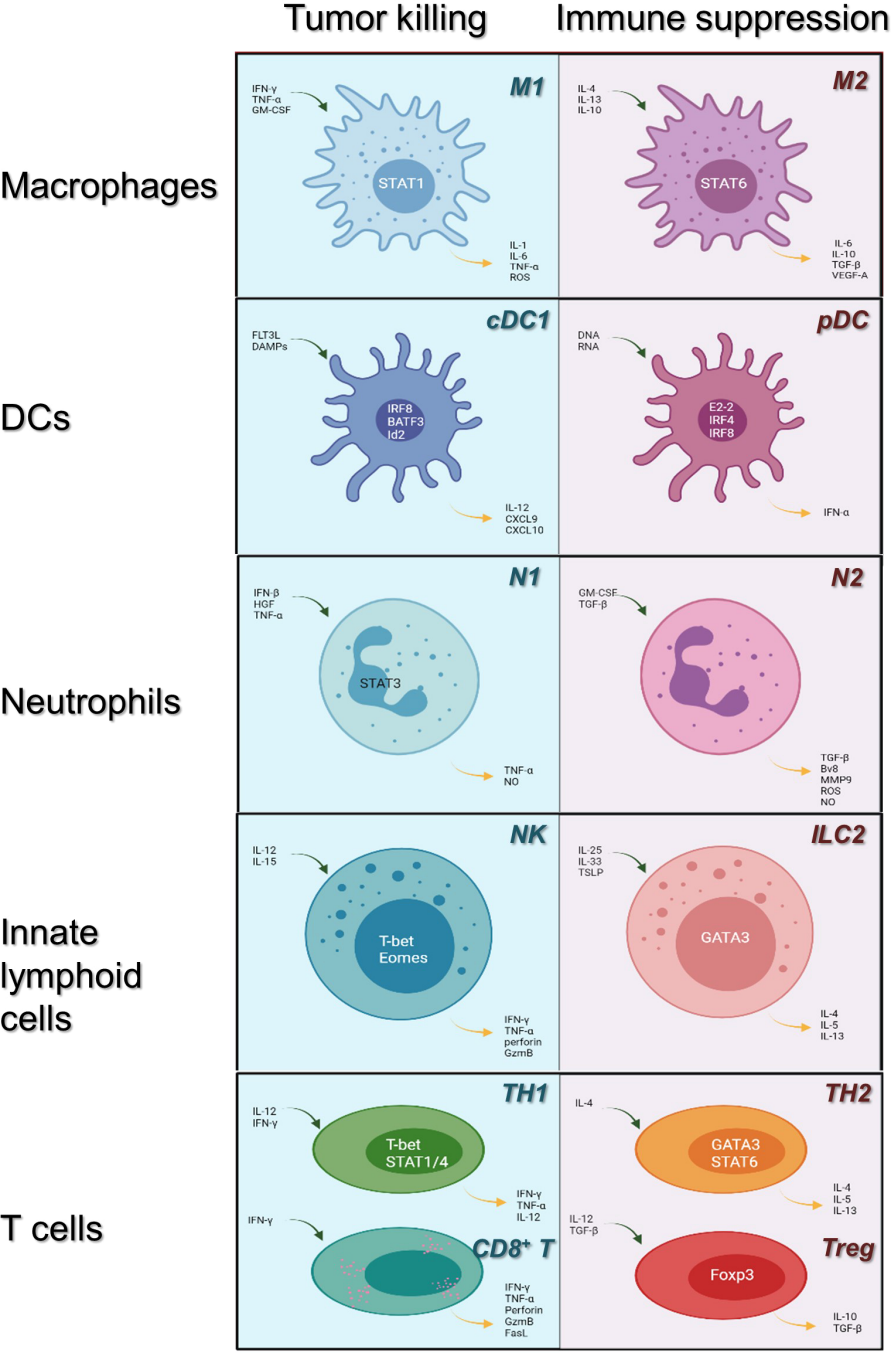
Immune cells in the tumor microenvironment: roles in tumor killing and immune suppression. Immune cells may evolve into antitumor or pro-tumor phenotypes in response to their microenvironment. Here, we review the category of the major innate immune cell lineages (in rows) based on their roles in tumor killing and immune suppression (light blue, left; pale red, right, respectively). Main transcription factors occupy the center of each cell; blue arrows indicate cytokines upstream of each phenotype, whereas yellow arrows indicate downstream cytokines. cDC1, conventional dendritic cells 1; pDC, plasmacytoid dendritic cells; NK, natural killer cells; ILC 2, innate lymphoid cell type 2; TH1/2, CD4^+^ T helper cell types 1 or 2; CD8+ T: CD8+ T cells; Tregs: CD4+ regulatory T cells.

Efficacious cancer treatment remains challenging due to chemoresistance and toxicity. Therefore, limited success can be achieved with traditional chemotherapy. Tumor cells induce TME to suppress antitumor immunity, and immunosuppressive cells and cytokines constitute the extrinsic factors of tumor drug resistance. Today, immunotherapy is regarded as a promising and revolutionary therapeutic option for multiple cancers and has received considerable attention. The discovery of cancer therapy through inhibition of negative immune regulation was recognized with the 2018 Nobel Prize. Detailed classification of the main tumor-infiltrating immune cell lineages is shown in **Figure 1**.

Human antibodies targeting immune checkpoint proteins are used to break immune tolerance and activate T cell responses. These antibodies are called ICIs and include cytotoxic T lymphocyte associated protein 4 (CTLA4), programmed death-1 (PD-1), and programmed death-ligand 1 (PD-L1) (128–130). A variety of methods such as adoptive T cell transfer (ACT), STING agonists, and cancer vaccines leverage the immune system to assis in recognizing and rejecting tumors. However, recent studies have highlighted that the TME can inhibit the functions of immune cells to favor immunological resistance and suppress antitumor effector functions, indicating the interwoven relationship between the TME and immunotherapy (131–133).

In addition, diverse strategies have been proposed to either enhance the function of antitumor effector cells or to dampen the protumor activities of immunosuppressive cells (134). In the following section, we will present a general review of current state-of-the-art immunotherapies as well as the obstacles that must be addressed to increase their efficacy.

### 3.1 Application of ICIs

To reactivate the immunological response of T cells and restore immune activity in the TME, a single or combined dose of ICIs inhibits the transmission of immunosuppressive signals, eventually contributing to the antitumor effect. Two types of ICIs have been approved by the FDA thus far: CTLA4 (ipilimumab) as well as PD-1 (nivolumab and pembrolizumab) or PD-L1 (atezolizumab) (135). Owing to the high response rates of prolonged duration among certain subsets of melanoma, non-small-cell lung cancer, and renal-cell carcinoma, the desire to establish new clinical trials for OS has increased (136–139). Of note, this enthusiasm should be moderated because of the hysteretic anti-OS drug testing meditated by ICIs. The insensitive effect for OS treatment has been revealed according to the preclinical studies showed in anti-PD-1 monotherapy (140). It is of great value to evaluate the role of chemoresistance to therapeutic ICIs in OS and to enhance the sensitivity of OS tissue to anti-PD-1 monoclonal antibodies. Therefore, more research is required to design successful endogenous antitumor activity and a prospective application to improve tumor immunogenicity. Significantly, the factors determining the remarkable efficacy of ICIs may include but are not limited to T cell intratumoral distribution, expression of PD-1/PD-L1, tumor antigenicity, and fitness of tumor-infiltrating T cells (127).

#### 3.1.1 PD-1/PD-L1 in OS

**Figure 2:**
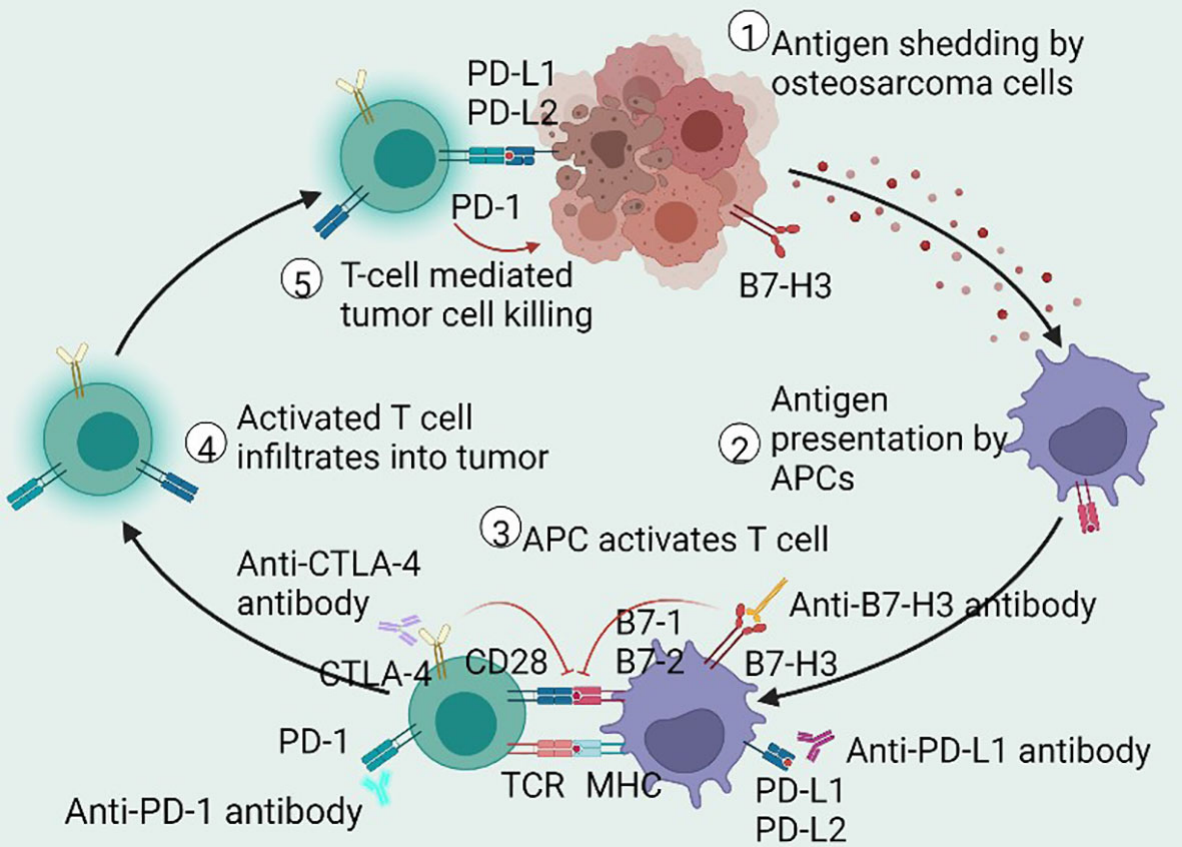
Schematic of antitumor immune cycle. The immune cycle starts from the production of neoantigens in dying or dead OS cells, endocytosed by APCs for presentation or cross-presentation on MHC. Then, the antigen-loading APCs migrate to the draining lymph nodes to activate antigen-specific T cells. Activated T cells then infiltrate the tumor cells to drive adaptive immune response and to restrain tumor growth. These antitumor immune responses are modified by immune checkpoint mechanisms. The interaction of PD-1 and PD-L1 inhibits intracellular signaling pathways on T cell activation, whereas CTLA-4 prompts inhibitory effects by competitively depriving CD28 ligand and mechanistically binding B7 molecules. Antibodies that affect ICIs may sustainably stimulate the antitumor immune response in patients with OS. OS, osteosarcoma; APCs, antigen-presenting cells; MHC, major histocompatibility complex; PD-1, programmed cell death receptor-1; CTLA-4, cytotoxic T lymphocyte-associated protein 4; B7-H3, B7 homolog 3; PD-L1/PD-L2, programmed cell death receptor-1/2 ligand; ICIs, immune checkpoint inhibitors.

PD-1 (CD279) is expressed on the surface of activated CD8+ T cells, B cells, and NK cells (141). The ligands of PD-1 are PD-L1 (CD274 or B7-H1) and PD-L2 (CD273 or B7-DC), which are typically expressed on the surface of APCs, tumor cells, and tumor-infiltrating lymphocytes (TILs) within the TME (141). The engagement of PD-1 and PD-L1/PD-L2 results in a negative signal for the inhibition of cytokine secretion and lymphocyte proliferation, interferes with the formation of immunological synapses, and inhibits T cell receptors (TCRs) (142, 143), resulting in an attenuated antitumor immune response (**Figure 2**).

Studies involving PD-1, PD-L1, and TIL expression in OS cell lines and tumor tissues are listed in **Table 5** (139, 144–151). According to a series of studies, in 15 patients with OS, biopsy samples demonstrated PD-1 and PD-L1 expression (47 and 53%, respectively) and metastases samples showed 40 and 47%, respectively, whereas resection samples showed no expression at all, indicating that biopsy or metastatic samples are most useful in determining whether PD-1 and PD-L1 are active (152). Using flow cytometry, PD-1 expression was measured in 56 OS patients and 42 healthy donors, revealing that PD-1 expression was significantly upregulated in both peripheral CD4+ and CD8+ T cells in OS patients (150). Furthermore, cases with metastasis had a higher proportion of PD-1 expression in CD4+ T cells (150), particularly within the lung (153). Moreover, researchers have suggested that in the stage III cases, the expression quantity of PD-1 on CD4+ T cells was significantly increased. PD-1 expression on CD8+ T cells varied with tumor stage, as it began to increase from stage II onward. These results (150) showing dysregulated PD-1 expression in patients with OS suggests its critical role in the development of this disease. Additionally, although PD-L1 expression in OS cell lines varies widely from low to high, doxorubicin-resistant OS cells seem to express higher PD-1 than that of non-resistant wild-type cells (154).

**Table 5:** Studies of PD-1/PD-L1 expression in OS.

Study	Samples	Detection techniques	Positive Expression of PD-1/PD-L1	Clinical guide
Chen et al. (2020) (139)	15 OS patients	IHC	Biopsy samples (PD-1 47% and PD-L1 53%); none in resections; metastases samples (PD-1 40% and PD-L1 47%)	Assessment of PD-1/PD-L1 in biopsy or metastatic specimens have clinical value in predicting therapeutic response.
Torabi et al. (2017) (144)	OS samples	Western blot	Positive PD-L1 expression	—
		qRT-PCR	More content of PD-1 mRNA	—
	26 OS samples	IHC	PD-1 detected in all tissue samples	—
Costa Arantes et al. (2017) (145)	9 oral OS patients of 13	IHC	High positive expression of PD-L1	No significant correlation of PD-L1 gene expression with clinicopathologic features.
Sundara et al. (2017) (146)	85 samples	IHC	Positive rate of PD-L1 is 27.8%	Higher expression of PD-L1 was detected in metastatic lesions (48%)
Koirala et al. (2016) (147)	Cell lines	Western blot	Positive rate of PD-L1 is 40%	Primary OS tumor expressing PD-L1 were more likely to contain cells that express PD-1.
		qRT-PCR	Positive rate of PD-L1 mRNA is 75% within 21 cell lines	—
	107 tissue samples	IHC, flow cytometry	Positive rate of PD-L1 mRNA is 67% within tumor specimens	Expression level of PD-L1 is connected with the presence of T cells, DCs and NK cells.
		Western blot	Positive rate of PD-L1 is 30% within patient samples	—
Lussier et al. (2015) (148)	16 patients	IHC	Positive rate of PD-L1 is 75% within the metastatic OS	Metastatic tumors can tolerize infiltrating T cells within TME by PD-L1 interactions
Chowdhury et al. (2015) (149)	15 OS patients of 115 pediatric tumors	IHC	Positive rate of PD-L1 expression is 47% among OS patients	Patients expressing PD-L1 showed distinctly better survival
Zheng et al. (2015) (150)	56 OS patients	IHC, flow cytometry	High expression level of PD-1 is detected in peripheral CD4+ and CD8+ T cell within OS patients	PD-1 is involved in tumor progression.
Shen et al. (2014) (151)	OS cell lines	qRT-PCR, IHC, flow cytometry	There is slightly higher PD-L1 expression of drug-resistant variants OS cell lines in comparison with that in parental cell lines	—
	38 patients with OS	qRT-PCR, IHC, flow cytometry	High PD-L1 expression level (23.7%)	—
			Intermediate PD-L1 expression level (50%)	Median survival time is 89 months at low levels of PD-L1 but is only 28 months at high levels of PD-L1.
			Low PD-L1 expression level (10.5%)	PD-L1 expression is distinctly related to TIL expression.
			Negative PD-L1 expression level	Pulmonary metastatic cases showed higher PD-L1 expression than that of the non-pulmonary metastatic lesions.

*OS, osteosarcoma; PD-1, programmed cell death receptor-1; PD-L1, Programmed cell death receptor-1 ligand-1; IHC, immunohistochemistry; qRT-PCR, quantitative real time polymerase chain reaction; DCs, dendritic cells; NK cells, natural killer cells; TME, tumor microenvironment; TILs, Tumor-infiltrating lymphocytes.

There have been promising results in preclinical OS mouse models where the PD-1 and PD-L1 pathways have been blocked. In a mouse model of metastatic OS, the function of T cells can be significantly activated by interactions with the PD-1/PD-L1 antibody *in vitro* and *in vivo*, consequently resulting in an increased survival rate (155). In a humanized mouse model, Zheng et al. (156) confirmed that nivolumab restrained OS metastasis by boosting CD4+ and CD8+ lymphocytes as well as the cytolytic activity of CD8+ T cells in the lung. This indicates that the PD-1 blockade effectively controlled OS pulmonary metastasis but did not affect primary lesions *in vivo*. When given sequentially and continuously, anti−PD−L1 combinatorial treatment (atezolizumab) with GD2− or HER2−BsAb enhanced T cell function *in vivo* and improved tumor control and survival time in the OS mouse model (157). Furthermore, Liu et al. (158) revealed that atezolizumab suppressed tumor proliferation and induced immune-independent apoptosis of OS by impairing intracellular mitochondria, resulting in increased ROS and cytochrome-c leakage, subsequently activating the Jun N-terminal kinase (JNK) pathway to give rise to apoptosis.

As for clinical trials, compared to the objective response rate (ORR) (18%) of the advanced soft tissue cohort, the ORR in the bone sarcoma cohort was 5% with 1PR/22 in OS within the open-label multicenter phase II trial of pembrolizumab (SARC028) (159). This study showed that the activity of the anti-PD-1 immunotherapy in bone sarcomas was limited because of the ineffective ORR. In another phase II trial in advanced OS (Norway/Rizzoli collaboration trial, NCT03013127), pembrolizumab was well-tolerated but only demonstrated minor clinically significant antitumor activity (160). We summarized the clinical trials using ICIs for patients with OS in **Table 6**.

**Table 6:** Clinical trials of immune checkpoints inhibitors for patients with osteosarcoma.

Clinical trial	Phase	Treatment	Intervention	Immunotherapy targets
NCT02301039 (SARC028)	II	Pembrolizumab	Pembrolizumab will be administered i.v. at 200 mg every 3 weeks	PD-1
NCT03013127	II	Pembrolizumab	Pembrolizumab 200 mg i.v. every 3 weeks for up to 35 cycles	PD-1
NCT05182164 (PEMBROCABOSARC)	II	Pembrolizumab + Cabozantinib	Pembrolizumab will be administered i.v on day 1 every 3 weeks (200 mg). Cabozantinib will be administered per OS once daily (40 mg)	PD-1
NCT02500797	II	Nivolumab	Patients receive nivolumab i.v over 30 minutes once every 2 weeks. Cycles repeat every 42 days for up to 108 weeks in the absence of disease progression or unacceptable toxicity. Patients who progress after 10 weeks on single agent nivolumab may elect to cross over to Arm II.	PD-1
NCT03628209	I/II	Nivolumab + Zacitidine	Participants will be treated with nivolumab i.v., 3 mg/kg on days 1 and 15 of each cycle. Phase I Dose Escalation - Dose level 1: NA. Dose level 2: 60 mg/m^2. Dose level 3: 75 mg/m^2. Phase II Expansion - Treated at recommended Phase II dose (RP2D).	PD-1
NCT04803877	II	Regorafenib + Nivolumab	Regorafenib 40 mg + 480 mg i.v. over 30 min every 28 days for patients aged 18 and older; regorafenib 20 mg + nivolumab 3 mg/kg (maximum dose 240 mg) will be administered i.v. over 30 minutes on day 1 and 15 of each 28-day cycle for subjects younger than 18 years;	PD-1
NCT02304458	I/II	Nivolumab + Ipilimumab	—	PD-1 + CTLA-4
NCT05302921	II	Nivolumab + Ipilimumab	Nivolumab and ipilimumab will be given on day 1 of 21-day cycles for cycles 1-4, followed by nivolumab alone on days 1 and 15 of 28-day cycles for cycles 5+. Patients will receive up to 13 cycles of therapy unless unacceptable toxicity or progression of disease.	PD-1 + CTLA-4
NCT05019703 (TACOS study)	II	Atezolizumab + Cabozantinib	Patients receive atezolizumab IV over 60 minutes on day 1 and cabozantinib PO QD on days 1-21.	PD-L1
NCT01445379	I	Ipilimumab	Ipilumumab given on day 1 of a 21-day cycle for 4 cycles, from cycle 5+	CTLA-4

*IV, Intravenous; OS, osteosarcoma.

#### 3.1.2 CTLA-4 in OS

CTLA-4 (CD152) is a transmembrane glycoprotein primarily expressed by T cells (161). In the immune cycle, T cells can be activated when antigens are presented to TCRs by MHC-I or MHC-II, which is amplified by a costimulatory signal in the form of the co-activating receptor CD28 binding to CD80 (B7–1) and CD86 (B7–2) expressed on antigen-presenting cells (APCs) (162). CTLA-4 can bind to CD80/CD86. Due to the greater affinity of CTLA4 to B7 proteins than to CD28, CTLA4 delivers inhibitory signals of T cell proliferation to downregulate immune responses by preventing the binding of CD28 with CD80/CD86 in the priming phase (135), as shown in **Figure 2**. CTLA4-mediated inhibitory signaling is complex and occurs within the lymph nodes, whereas it is generally in the peripheral tissue where PD-1-mediated inhibitory signaling takes place. Although CTLA4 and PD-1 signals inhibit the activity of AKT signaling pathways, the targeted signaling molecules are disparate. CTLA4 signaling dampens T cell activation pathways by interacting with IL-2, serine/threonine phosphatase PP2A, and SHP2, which directly dephosphorylates CD3ζ (163). In addition, recent studies have revealed a significant association between CTLA4 genetic polymorphisms and susceptibility to OS (164, 165).

In another notable study, scientists tested combinatorial anti-CTLA-4 and anti-PD1/PD-L1 therapy in an animal model of metastatic OS, showing that this regimen resulted in the complete control of tumors and immunity to further tumor inoculation (166), suggesting that such therapy may be more beneficial than stand-alone monotherapy. In addition, the CTLA-4 antibody, which combines with dendritic cells, can decrease the level of CD4+ regulatory T cells (Tregs) and increase the concentration of cytotoxic T cells in metastatic OS mice for tumor suppression (167, 168). In a phase I trial with ipilimumab, four of 33 patients with advanced pediatric solid tumors (including eight OS patients) confirmed stable disease and two patients had unconfirmed stable disease by standard RECIST criteria, indicating that there is no objective tumor regression under the treatment with ipilimumab (169). Recent meta-analysis showed that CTLA-4 is significantly associated with OS risk and may play a crucial role in carcinogenesis of OS (164, 170). A full description of the clinical trials is provided in **Table 6**.

In summary, although the application of PD-1 or PD-L1 antibodies showed promising outcomes in suppressing tumor growth in an OS mouse model, the effects of ICIs had limited therapeutic benefit for patients with OS in clinic trials. Unfortunately, there have been no current breakthroughs in clinical trials involving new drugs developed for this dilemma. However, mifamurtide was shown to improve overall survival in a phase III trial (70). Moreover, mifamurtide would promote immune cell to infiltrate into OS metastases, consequently improving the efficacy of anti-PD-1 antibodies (171).

#### 3.1.3 T cell immunoreceptor with Ig and ITIM domains in OS

T cell immune checkpoint molecules may be prospective immunotherapeutic targets for tumor therapy. Currently, anti-T cell immunoreceptor with Ig and ITIM domains (TIGIT) therapies are considered curative checkpoint markers because of their potential to treat hepatocellular carcinoma and breast cancer by modulating CD8+ T cells, Tregs, and NK cells (172, 173). Wang et al. showed that macrophage M1 types, which are highly infiltrated in metastatic cases, could predict the overall survival and disease-free survival of OS, which would be positively connected to immune checkpoints PD-L1, CTLA4, and TIGIT (174). Zhou et al. (175) revealed that TIGIT was widely expressed in CD8+ T, CD4+ T, and NK cells, and that Tregs showed high immunoinhibitory molecules involving TIGIT in OS through bioinformatics analysis, indicating that TIGIT blocking may be a promising avenue for OS treatment. In addition, peripheral blood CD3+ T cells were isolated from OS tissues with high and low infiltrated TIGIT+CD3+ T cells respectively for the detection of cytotoxic activities of the CD3+ T cells. These results (175) suggest that the TIGIT-blocking antibody substantially reinforced the cytotoxicity of CD3+ T cells to promote the death of OS cells, demonstrating the possibility of TIGIT inhibition for future OS therapies.

#### 3.1.4 Indoleamine 2,3-dioxygenase in OS

The beginning and rate-limiting stages of the kynurenine pathway in the metabolism of the essential amino acid tryptophan are catalyzed by the intracellular enzyme indoleamine 2,3-dioxygenase (IDO) (176). The biological function of IDO involves the protection of tumor cells by inhibiting attacks from T cells (177). High expression of IDO was observed in multiple tumors, such as pulmonary, colorectal, and melanoma (178–180), indicating a clinical adverse prognostic factor (179). Liebau et al. (181) demonstrated for the first time that IDO was activated by IFN-γ in four human OS cell lines and concluded that IDO was highly expressed in human OS cells. Urakawa et al. also confirmed these findings (182). Furthermore, the authors revealed that elevated IDO expression in OS was associated with metastasis and a poor clinical outcome in patients by univariate analysis.

However, the multivariate analysis has been particularly disappointing, showing that there was no discernible link between IDO expression and metastasis-free survival or overall survival. Taken together, IDO may be a reliable and promising prognostic predictor and has the potential to become a novel molecular target in the therapy for OS. At present, more research is required to undertake the challenges of improving immunotherapy efficacy.

### 3.2 Adoptive T cell transfer in OS

ACT refers to collecting innate T cells from cancer patients, expanding or genetically engineered them *ex vivo*, and retransferring them back into the patient with the intent to specifically kill cancer cells. There are currently three major modalities of ACT: TILs, engineered T cell receptor (TCR) T cells, and chimeric antigen receptor (CAR) T cells. Among these three categories, CAR T cell therapy has facilitated transformational advancements in the management of cancer treatments. For instance, the impressive results of CAR-T therapy trials prompted its usage by the FDA in refractory large B cell lymphoma and acute lymphoblastic leukemia (183, 184). For OS, clinical trials have been performed with several promising target antigens. For instance, HER2-CAR T cells proved to be therapeutic for OS through xenograft *in vitro* and *in vivo* models. Phase I/II clinical trials were conducted by applying CAR-T therapy in patients with relapsed/refractory HER2-positive sarcoma, with 16 enrolled OS patients (84%) (185). Out of these 16 patients, none (100%) had an objective response. Three patients (19%) had stable disease for 12–15 weeks while 11 patients (69%) had progressive disease. The value of this study is that it confirms that dose-limiting toxicity is not observed in HER2-CAR T cell reception, which prepares for additional studies that combine ACT with other anticancer treatments to enhance their expansion and persistence. Presently, breakthrough successes have been achieved in the clinic for hematological malignancies and have gained interest in developing ongoing trials to extend CAR-T therapy application to solid tumors as well as its usage beyond cancer. Additionally, T lymphocytes expressing CAR or TCR can recognize a wide range of antigens and are not restricted to tumor-specific antigens due to retaining their endogenous TCR expression (127). Therefore, autoimmune diseases or other immune-mediated hyper-responses may be triggered in patients undergoing ACT.

### 3.3 Targeting NK cells in OS

A strategy that incorporates NK cells into OS treatment represents a promising immunotherapeutic approach to boost tumoricidal properties. Although activated NK cells can express PD-1 (186) and CTLA4 (187), more research is required to determine whether ICIs directly affect NK cells. It has been reported that anti-PD-1 treatment can re-engage NK cell antitumor responses in multiple myeloma (188). Furthermore, blockade of CTLA4 or release of cytokines can overcome the stagnancy in NK cell antitumor responses (161). Clinical trials (189) have shown that NK cells may have the potential to attack and eliminate cancer cells for OS prevention and treatment response. Compared to normal controls, the quantitative observation of lower-level NK cell defects in peripheral blood of patients with OS indicated the regulatory role of NK cells in human autoimmunity and OS tumor development. NK cell antitumor activity is determined by reactivity and inhibition of NK cells and their engagement by cognate ligands toward target tumor cells (190). Metastatic and primary OS cells are susceptible to activated/expanded NK cell lysis both *in vivo* and *in vitro*, which relies on heterogeneous interactions between the NK group-2 member D (NKG2D) receptor and NKG2D ligands (NKG2DL) (191). In other words, NK cells can kill OS cells, including the tumor-initiating cell (TIC) compartment, in an NKG2D–NKG2DL dependent manner. In addition, the NK cell-derived NK-92 cell line has been genetically modified to express CARs that target both hematological malignancies and solid tumor antigens in preclinical and clinical trials (192), such as GD2 on neuroblastomas (193) or HER2 on neoplasms (194).

However, despite these conflicting results, several hindrances need to be overcome to maximize the curative effects of NK cell-based immunotherapies. Such obstacles include patients needing to be injected with a large number of cells, the lack of cellular memory, poor NK cell infiltration of solid tumors, limited expansion *in vivo*, and systemic toxicity of cytokines such as IL-2 (195, 196). Hence, to optimize NK cell infiltration and performance in solid tumors, it is imperative that strategies be developed to address these issues.

## 4 Discussion

OS is the most common malignant bone tumor. Although OS is sensitive to some chemotherapeutic drugs, cancer cells may develop chemoresistance. Although advances in neoadjuvant chemotherapy and their rapid and wide applications have a crucial impact on the overall survival rate of patients with OS, their overall survival rate has not significantly improved over the last 30 years. Similarly, the prognosis of patients with metastatic or recurrent OS remains poor, with an overall five-year survival rate of 20% (197). Current standard treatment for OS therapy is the delivery of chemotherapeutic agents such as high-dose methotrexate, doxorubicin, and platinum salts. However, clinical outcomes from chemotherapy have been reported to be unsatisfactory in recent studies. To date, there have been no obvious breakthroughs in clinical trials of new drugs developed for this dilemma. Furthermore, many clinical trials have found that the efficacy of most promising targeted therapies is very poor and far below expectations (198–202).

The main reasons for the lack of development of OS therapy include tumor heterogeneity, chemoresistance, and the lack of discovery of tumor-specific antigens (TSA) in OS. Some studies have emphasized that the TME is involved in the proliferation and migration of cancer cells (199, 203). Despite vital improvements made in preclinical trials, many clinical trials targeting the TME to suppress tumor growth or improve drug resistance have failed to show promising efficacy in multiple cancers. The only exception is immunotherapy, including the usage of ICIs (15). In fact, most anticancer therapies act on immune regulatory factors that comprise part of the TME. The immune microenvironment should also be regarded as a clinical treatment option. Furthermore, these cells and molecules that constitute the OS microenvironment may improve the chemoresistance and enrich potential therapeutic targets for OS therapy, such as blood vessels, T cells, and macrophages (198, 204–206). Given the barriers of OS treatment involved in chemoresistance, novel therapeutic approaches to treat OS is urgently needed. The immune system is a significant part of the OS microenvironment, in which cytokines are closely related to the development and dynamic balance of bone cells. As a novel antitumor model, immunotherapy benefits from the immune system in a subtle way to improve anticancer treatment efficacy. Finding biomarkers that can be used to predict responses is a leading difficulty in immunotherapy but would help determine the best possible treatment options. Multiple tumor immune phenotypes (PD-1 or PD-L1 expression), somatic genomic characteristics (mutational burden and microsatellite instability), the gut microbiome (207), and the HLA class I genotype (208) have all been proposed as predictors of responses to checkpoint inhibitors.

### 4.1 Mechanisms of unsatisfactory effects of ICIs against OS

Though ICIs such as PD-1 or PD-L1 showed promising results in preclinical research, OS showed minor tumor regression with the usage of ICIs based on the current clinical trial results listed in **Table 6**. The main reason for the unsatisfactory effects of PD-1 antibodies can be summarized into four points: 1) insufficient immunogenicity of TSA: the lack of highly immunogenic TSA resulting in the inability of T cells to recognize tumor cells. A higher burden of nonsynonymous mutations with durable clinical benefit displayed in patients with non-small cell lung cancer treated by anti-PD-1 through exome sequencing (209). Therefore, we hypothesized that tumors with high mutational burden have a higher probability of producing more neoantigens with sufficient immunogenicity to induce antigen-specific T cell responses; 2) dysfunction of MHC: variable PD-L1 expression and frequent loss of MHC I facilitates immune evasion of OS cells. The mutation of beta 2-microglobulin (β2-GM) led to dysfunctional antigen presentation of HLA I complexes, which were active in the MHC I pathway, resulting in the weakening cytotoxicity of T cells (210); 3) paucity of CD8+ T cells: shortage of CD8+ T cells upregulated multiple inhibitory receptors, such as CTLA-4, PD-1, T cell immunoglobulin, mucin domain 3 (TIM-3), T cell immunoglobulin, TIGIT, and lymphocyte-activation gene 3 (LAG-3), as well as by producing immunosuppressive cytokines or other soluble factors (211, 212); 4) inhibition of the TME: immunosuppressive mechanisms in the TME involve the suppressive action of Tregs, MDSCs, TAMs, or other undefined cells, and the specific mechanism of these cells as mentioned above. Furthermore, cytokines and tumor-derived chemokines would also meditate drug resistance by recruiting immunosuppressive cells into the TME (213).

### 4.2 Future perspectives

Targeting components of the TME, such as immune cells, immunosuppressive cytokines, and inhibitory receptors of T cells may be a novel therapeutic approach to improve the dilemma of drug-resistance and the unconspicuous OS tumor recession. Although the significant breakthroughs toward improving outcomes of TME target therapies have been made *in vitro* and *in vivo*, promising efficacy in human OS patients remains to be seen. In multiple malignancy, immunotherapy is the jewel in the crown because of its the unique exception of feeble TME targeting therapy (15). Therefore, immunotherapy involving ICIs could be an effective and alternative tact to avoid the hindrance faced in OS treatments. For the success of OS immunotherapy, it is necessary to expound the mechanism of immunosurveillance, confirm TSA for OS, and conduct collaborative multicenter research.

Several biological features of OS imply that modulation of the immune response regulation may be beneficial. However, nuances within the specific TME and the complexity of the immune system make it an extremely challenging work. As seen with conventional chemotherapy drugs, tumors utilize multiple pathways to resist immunotherapy, suggesting that combinatorial approaches targeting multiple pathways will be explored to achieve robust responses. Chemotherapy, radiotherapy, tumor-vaccines, and ICIs, or compatibility with ACT, may yield meaningful clinical benefits. Additionally, in the OS microenvironment, Tregs, TAMs, and MDSCs could play crucial roles in immunoreaction with overactivated inhibitory receptors including PD-1, CTLA-4, and TIGIT. In order to develop targeted immunotherapies through utilizing those immunologic markers in intratumoral microenvirenments, we must better understand and characterize the OS immune system. Other adults strategies explored, including the combination of PD-1 agents and IDO since IDO has been shown to inhibit T-cell proliferation and induce Tregs, among other immunosuppressive properties. The ubiquitous expression of IDO in primary OS may make the combinatorial strategy more attractive for OS treatments.

A more comprehensive understanding of the mechanisms of resistance is likely to be required for the development of effective therapies for patients with OS, identifying predictive biomarkers to help guide the appropriate usage of these treatments, as well as developing rational combinatorial treatments to overcome such resistance. Despite many challenges, there is hope that immunotherapy will lead to breakthroughs that will revolutionize OS therapy.

## Author contributions

LY, JZ, and YL substantially contributed to the conception, drafting, editing, and final approval of this manuscript. All authors contributed to the article and approved the submitted version.

## Conflict of interest

The authors declare that the research was conducted in the absence of any commercial or financial relationships that could be construed as a potential conflict of interest.

## Publisher’s note

All claims expressed in this article are solely those of the authors and do not necessarily represent those of their affiliated organizations, or those of the publisher, the editors and the reviewers. Any product that may be evaluated in this article, or claim that may be made by its manufacturer, is not guaranteed or endorsed by the publisher.

## Abbreviations

ACT, adoptive T cell transfer; BIM, BH3-only protein, a mediator of apoptosis; CAR, chimeric antigen receptor; CSCs, cancer stem cells; CSF1R, colony-stimulating factor 1 receptor; CTL, cytotoxic T cells; CTLA4, cytotoxic T lymphocyte associated protein 4; EMA, European Medical Agency; EMT, epithelial-mesenchymal transition; HIFs, hypoxia-inducible factors; HRE, hypoxia response element; ICIs, immune checkpoint inhibitors; IDO, indoleamine 2,3-dioxygenase; IFNγ, interferon-gamma; iNOS, inducible nitric oxide synthase; JNK, Jun N-terminal kinase; LAG-3, lymphocyte-activation gene 3; MDSCs, myeloid-derived suppressor cells; Met, metformin; NF-κB, nuclear factor-kappa B; ORR, objective response rate; OS, osteosarcoma; OXPHOS, decreased oxidative phosphorylation; PD-1, programmed death-1 and; PD-L1, programmed death-ligand 1; PFS, progression-free survival; PGE2, prostaglandin E2; PMN-MDSCs, polymorphonuclear MDSCs; ROS, reactive oxygen species; SDF-1, stromal cell-derived factor-1; SIRPα, signal regulatory protein α; STAT3, signal transducer and activator of transcription 3; TAMs, tumor-associated macrophages; TCR, T cell receptor,; TCRs, T cell receptors; TIC, tumor-initiating cell; TIGIT, anti-T cell immunoreceptor with Ig and ITIM domains; TILs, tumor-infiltrating lymphocytes; TME, tumor microenvironment; Tregs, regulatory T cells; TSA, tumor-specific antigens; VCAM-1, vascular cell adhesion molecule 1; VEGF, vascular endothelial growth factor.
